# The Effect of a Maturing Antiretroviral Program on Early Mortality for Patients with Advanced Immune-Suppression in Soweto, South Africa

**DOI:** 10.1371/journal.pone.0081538

**Published:** 2013-11-28

**Authors:** Kennedy N. Otwombe, Fatima Laher, Thandeka Tutu-Gxashe, Glenda Gray, Lerato Mohapi

**Affiliations:** Perinatal HIV Research Unit, University of the Witwatersrand, Johannesburg, Gauteng, South Africa; University of New South Wales, Australia

## Abstract

**Objective:**

We hypothesize that time to initiate care and maturity of a treatment program impact on outcome of severely immuno-compromised patients with higher risk of mortality.

**Design:**

We conducted a retrospective cohort analysis at the Perinatal HIV Research Unit Adult ART clinic, Soweto, South Africa.

**Methods:**

Eligibility criteria for this analysis were: attendance for minimum one visit between August 2004 and August 2010, age >18 years, CD4 count < 50 cells/mm^3^ and ART-naïve at screening. We followed participants up to one year after ART initiation. We defined years 2004-2007 and 2008-2010 as the early and late eras respectively. Chi-square test and survival analysis methods were used for mortality comparisons between eras.

**Results:**

Of 2357 patients eligible for antiretroviral treatment, 395 (17%) had CD4 counts < 50 cells/mm^3^ and ART-naïve at screening. Overall 261 (66%) were women. Patients had similar median age (35 vs. 33.5 years, p=0.08), time to HAART initiation (7 days, p=0.18) and baseline CD4 count (20 vs. 23 cells/mm^3^, p=0.5) between eras. Overall 63 (16%) patients died in their first year of treatment (2 per 100 person-months) and the main cause of death was tuberculosis (n=23, 37%). The proportion of deaths (52/262 vs. 11/133, p=0.003) and time to death from enrolment (logrank p=0.04) were significantly different between eras.

**Conclusion:**

Mortality decreased as the ART program matured in Soweto while time to initiation of treatment remained similar in both eras. Because ART guidelines were consistent during both eras, it is possible that with time, management of patients improved as expertise was gained.

## Introduction

The provision of free antiretroviral therapy (ART) services in sub-Saharan Africa has increased the number of people accessing treatment, consequently increasing life expectancy[1-3]. Unfortunately the triumphs of ART programs are eroded by early mortality rates on treatment observed especially in patients who present with severe immune compromise[4-7]. 

South Africa has the largest number of HIV-infected people and the largest ART program worldwide. Many patients present to the national treatment program at an advanced state of immune compromise: in one program in Khayelitsha, South Africa, the median CD4 count at entry was 43 cells/ul[8]. Multiple issues make the management of individuals with advanced immune suppression more difficult especially in a resource-constrained healthcare system: the increased risk of opportunistic illnesses which may be both difficult and expensive to diagnose and treat, the increased risk of immune reconstitution inflammatory syndrome, and the timing of ART initiation in the context of opportunistic disease. 

Lower CD4 counts signify advancing immune compromise and are associated with an increased risk of morbidity and mortality[6,9-11]. CD4 counts less than 50cells/mm^3^ are associated with a higher risk of opportunistic illnesses such as Pneumocystis jirovecii pneumonia (PJP), cryptococcal meningitis, cytomegalovirus retinitis, central nervous system lymphoma and deaths from tuberculosis[12]. 

Many individuals with advanced immune compromise present after they develop an acute opportunistic illness, and timing ART initiation in this context can be delicate when considering the risks of drug-drug interactions, additive adverse events, pill burden and immune reconstitution inflammatory syndrome. In more recent years however, evidence has shown that early ART initiation in the presence of an opportunistic infection can reduce disease progression and mortality[13-16]. 

The first South African ART guideline was published in 2004, and it remained in effect until 2010. During 2004-2010, ART initiation was permitted at CD4 cell count less than 200 cells/mm^3^. Additionally, adults with CD4 counts less than 50 cells/mm^3^ were targeted as a group for fast-track ART initiation within two weeks. National guidelines on timing of ART initiation for tuberculosis patients were that those with CD4 counts less than 50 cells/mm^3^ should be initiated within 2 weeks, and those with higher CD4 counts should be started within 2 months[17,18]. 

Our study focuses on those who presented for care with advanced immune compromise i.e. CD4 counts less than 50 cells/mm^3^, to assess the implementation of the national guideline and provide information on mortality outcomes.

## Methods

### Study design

This retrospective cohort analysis investigated median time to ART initiation and mortality in adult patients with screening CD4 count < 50 cells/mm^3^.

### Study setting

The study was conducted at the adult HIV Treatment Access clinic at the Perinatal HIV Research Unit (PHRU), Soweto, South Africa. The clinic has received funding from USAID/PEPFAR since 2004. It was accredited in 2010 by the National Department of Health as a Comprehensive Care Management and Treatment (CCMT) clinic. By the end of August 2010, 2357 HIV-infected individuals had been enrolled to receive treatment and care at the PHRU Treatment Access clinic.

### Patients

Eligibility criteria for this study were: attendance at the PHRU Treatment Access clinic for at least one visit between August 2004 and August 2010, CD4 count < 50 cells/mm^3^ at screening, 18 years or older and ART-naïve at screening (note: women who used short-course ART for prevention of vertical transmission were not excluded).

### Definitions

Screening was defined as the first clinic visit. Time to ART initiation was defined as the number of days between screening and ART initiation. Delayed ART initiation was defined as ART initiation beyond (but not including) 14 days. The early era was defined as patients screened and initiated between August 2004-2007 and the late era was defined as those patients screened and initiated between August 2008-2010. There were no treatment guideline changes between the two eras.

### Outcomes

We assessed two main outcomes within the first year of treatment: treatment initiation and mortality. 

### Measures

We conducted medical record reviews that focused on the time period between screening and one year post ART initiation. The following variables were collected: gender; date of birth; medical history including referral source, date of screening, CD4 count at screening, date of ART initiation, date of death or date of one year visit (whichever occurred first). 

Reasons for delayed initiation (defined in this analysis as time to initiation more than 14 days) were collated from medical records and classified into: co-morbid illnesses including hospitalisations; patient-related factors; institution-related and other reasons. Causes of death were collated from medical records which included hospital records where applicable or verbal autopsies from patient family members.

### Ethical approval

Written consent was obtained from each participant for their information to be stored in the clinic database and used for research purposes. Ethical approval for this study was obtained from the University of the Witwatersrand Human Research Ethics Committee (Medical).

### Statistical analysis

Continuous data was analysed descriptively through medians and interquartile ranges overall and by gender. Data was further split into the time periods 2004-2007 and 2008-2010 and continuous variables were compared non-parametrically using the Kruskall-Wallis test. Similarly comparisons were made between those initiating treatment within and after 14 days. Categorical comparisons between gender and eras were performed by chi-square analysis.

Kaplan-Meier methods and the log-rank p-value were used to compare time to death from enrolment and time of starting ART by gender and the periods 2004-2007 and 2008-2010. Univariate and multivariate Cox regression analysis controlling for gender, age and baseline CD4 count were used to determine predictors of mortality overall and in both eras. Hazard ratios, their 95% confidence intervals and p-values were used to determine significance. All statistical analysis was performed using SAS 9.3 and assuming a two-sided level of confidence.

## Results

**Figure 1:**
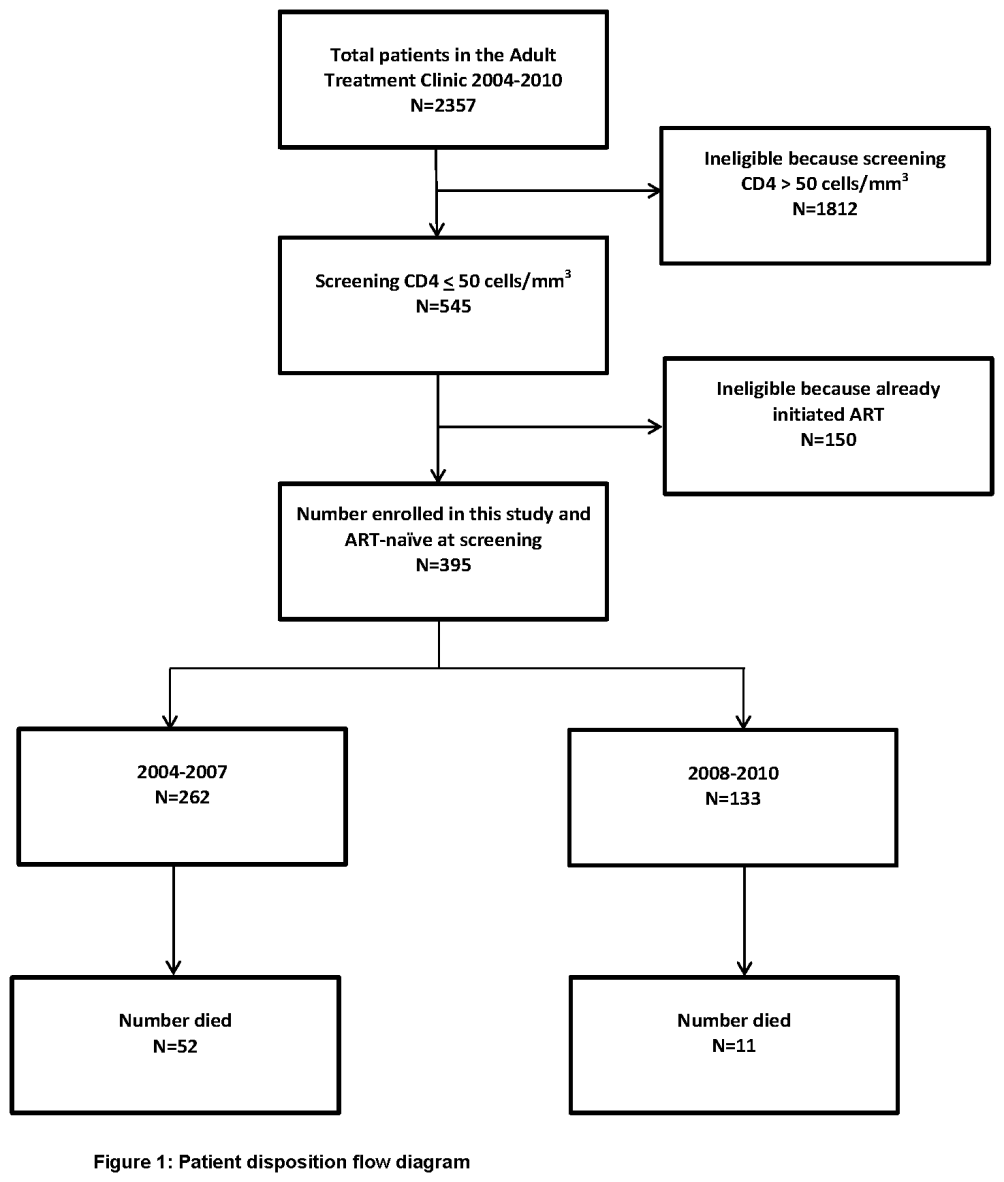
Patient disposition flow diagram.

A total of 2357 patients registered to receive treatment in the adult Treatment Access clinic at PHRU, in the six year period between August 2004 and August 2010. Of these, 395 (17%) met eligibility criteria for our analysis (Figure 1). Of those enrolled in this study, 262 and 133 were in the periods 2004-2007 and 2008-2010 respectively. Most of the participants were women (n=261, 66%). The median age at enrolment for men was 36.9 (IQR: 31.0-41.5) and 33.5 (IQR: 27.9-38.3) years for women (p-value < 0.0001). Males were significantly older than females at enrolment in both periods. The overall median CD4 count and days to ART initiation were similar between males and females (Table 1). The median (IQR) duration of follow-up was 11.1 months (5.6-12.1); it was 11.2 (10.4-12.6) in era one and 10.0 (0.9-11.4) in the second era. No participants were lost to follow-up in the one year follow-up duration of the study; the only terminations were from deaths.

**Table 1:** Patient characteristics at enrolment into the treatment access program.

**Variable**	**Overall**	**Males**	**Females**	**P-Value**
**Overall**				
Number enrolled	395	134	261	
Median age in years at screening (IQR)	34.4 (29.3-39.8)	36.9 (31.0-41.5)	33.5 (27.9-38.3)	<.0001
Median CD4 count at screening (IQR)	22.0 (8.0-37.0)	23.5 (9.0-38.0)	20.0 (8.0-36.0)	0.4192
Median days to ARV initiation (IQR)	7.0 (6.0-12.0)	7.0 (6.0-11.0)	7.0 (6.0-13.0)	0.7335
**2004-2007**				
Number enrolled	262	86	176	
Median age in years at screening (IQR)	35.0 (29.7-40.3)	36.7 (31.4-41.1)	34.0 (28.2-39.4)	0.0094
Median CD4 count at screening (IQR)	20.0 (8.0-36.0)	19.5 (7.0-37.0)	20.0 (8.0-35.5)	0.9723
Median days to ARV initiation (IQR)	7.0 (6.0-11.0)	6.0 (5.0-8.0)	7.0 (6.0-13.0)	0.0645
**2008-2010**				
Number enrolled	133	48	85	
Median age in years at screening (IQR)	33.5 (28.8-38.9)	37.6 (31.0-43.7)	32.4 (27.6-35.9)	0.0016
Median CD4 count at screening (IQR)	23.0 (10.0-38.0)	25.5 (11.5-40.0)	22.0 (9.0-37.0)	0.2203
Median days to ARV initiation (IQR)	7.0 (6.0-13.0)	9.0 (7.0-17.5)	7.0 (6.0-12.0)	0.0743

A total of 323 (82%) patients initiated treatment within 14 days of enrolment. There was no significant difference in the ages of those initiating ART within and after 14 days and by gender (Table 2). The median time of initiating treatment was 6 (IQR: 5-8) and 28 (IQR: 18-62.5) days (p<0.0001) for those initiating within and after 14 days respectively. The median baseline CD4 count was similar overall and in both periods in those initiating within and after 14 days. Further comparison between periods is presented in Table 3.

**Table 2:** Time of initiating treatment.

**Variable**	**Initiate ARVs within 14 days of screening**	**Initiate ARVs after 14 days of screening**	**p-value**
**Overall**			
Number (%)	323 (82)	72 (18)	
Age (IQR)	34.5 (29.7-40.5)	33.8 (28.1-37.9)	0.32
Gender			
Male (%)	110 (34)	24 (33)	0.99
Female (%)	213 (66)	48 (67)	
Median days to initiation (IQR)	6 (5-8)	28 (18-62.5)	<0.0001
Median baseline CD4 (IQR)	21 (8-37)	24 (10-35)	0.84
Deaths (%)	49 (15)	14 (19)	0.38
**2004-2007**			
Number (%)	218 (83)	44 (17)	
Age (IQR)	35.1 (30.3-40.5)	34.4 (28.2-37.9)	0.47
Gender			
Male (%)	76 (35)	10 (23)	0.16
Female (%)	142 (65)	34 (77)	
Median days to initiation (IQR)	6 (5-7)	28 (20-81.5)	<0.0001
Median baseline CD4 (IQR)	19.5 (7-35)	24 (11-36.5)	0.67
Deaths (%)	41 (18)	11 (25)	0.41
**2008-2010**			
Number (%)	105 (79)	28 (21)	
Age (IQR)	33.5 (29.0-39.9)	32.9 (28.1-37.8)	0.60
Gender			
Male (%)	34 (32)	14 (34)	0.97
Female (%)	71 (68)	14 (66)	
Median days to initiation (IQR)	7 (6-10)	38.5 (17.5-49.5)	<0.0001
Median baseline CD4 (IQR)	23 (10-39)	30.5 (9-34)	0.78
Deaths (%)	8 (8)	3 (11)	0.70

**Table 3:** Comparison of variables between eras.

	**Overall (N=395)**	**2004-2007 (N=262, 66%)**	**2008-2010 (N=133, 34%)**	**p-value**
**Median Age in years (IQR)**	34.4 (29.3-39.8)	35.0 (29.7-40.3)	33.5 (28.8-38.9)	0.08
**Gender**				
Male (%)	134 (34)	86 (33)	48 (36)	0.57
Female (%)	261 (66)	176 (67)	85 (64)	
**Median days to HAART initiation**	7 (6-12)	7 (6-11)	7 (6-13)	0.18
**Initiate ARV within 14 days**				
Yes (%)	323 (82)	218 (83)	105 (79)	0.3
No (%)	72 (18)	44 (17)	28 (21)	
**Proportion initiating within 14 days (%)**	323 (82)	218 (83)	105 (79)	0.3
**Median baseline CD4 (IQR)**	22 (8-37)	20 (8-36)	23 (10-38)	0.25
**Deaths (%)**	63 (16)	52 (20)	11 (8)	0.003
**Hospitalized**	22	16	6	0.5

In total, 63 (16%) patients died within one year of ART initiation at a rate of 2 (CI: 1.6-2.6) cases per 100 person-months (Table 2). There was no statistically significant difference in the proportion of deaths between those who initiated ART within versus after 14 days (15% vs. 19% respectively, p=0.38).Similarly the proportion of deaths was similar in those initiating within and after 14 days in both periods. There was a significantly (p=0.003) higher number of deaths in the period 2004-2007 (52, 20%) compared with 2008-2010 (11, 8%) (Table 3). The overall mortality rate was 2 (CI: 1.6-2.6) per 100-person months while it was 2 (CI: 1.5-2.6) and 1 (CI: 0.6-1.8) in 2004-2007 and 2008-2010 respectively. A total of 38 (60%) deaths occurred within the first three months of initiating treatment; 29 (56%) in 2004-2007 and 9 (82%) in 2008-2010 (p=0.11). At 6 months after initiating treatment, 42 and 10 deaths occurred in the first and second periods respectively (p=0.42).

The progression to death from enrolment was significantly higher in the first period compared to the second period (logrank p-value=0.04) while there was no difference in the overall time to death by gender (logrank p-value=0.2). There was no difference in the time to death from initiation of treatment stratified by gender (logrank p-value=0.2), period (logrank p-value=0.13) and time of ART initiation (logrank p-value=0.27).

In the overall multivariate Cox regression analysis (Table 4) adjusting for gender, age and baseline CD4 count, only those who had been hospitalized (HR: 3.896, CI: 1.935-7.847) predicted mortality. In the multivariate analysis stratified by period and adjusting for gender, age and baseline CD4 count, hospitalization (HR: 3.551, CI: 1.684-7.488) remained a significant predictor of mortality in the period 2004-2007. In the period 2008-2010, none of the variables (gender, age, time of ART initiation and hospitalization) predicted mortality. 

**Table 4:** Overall Predictors of mortality from HAART initiation.

**Variable**	**Univariate**		**Multivariate**	
	**HR (CI)**	**p-value**	**HR (CI)**	**p-value**
**Gender**				
Male	1.392 (0.840-2.307)	0.1989	1.538 (0.919-2.573)	0.1010
Female	Ref		Ref	
**Age**				
> 34	1.082 (0.660-1.774)	0.7557	0.925 (0.553-1.546)	0.7650
<= 34	Ref		Ref	
**Baseline CD4**	0.982 (0.965-0.998)	0.0323	**0.982 (0.965-0.999)**	**0.0374**
**Time of ART Initiation**				
Within 14 days	Ref		Ref	
After 14 days	1.398 (0.772-2.533)	0.2686	1.438 (0.788-2.625)	0.2369
**Hospitalized**				
Yes	4.119 (2.092-8.107)	<.0001	**3.896 (1.935-7.847)**	**0.0001**
No	Ref		Ref	
**Period**				
2008-2010	Ref		Ref	
2004-2007	1.652 (0.862-3.168)	0.1307	1.527 (0.785-2.972)	0.2125

Causes of death were attributed to tuberculosis infection (n=23, 37%; 15 in first era and 8 in the second, p=0.9075) , unknown causes (n=16, 25%), gastroenteritis (n=7, 11%), respiratory tract infections (n=5, 8%), cardiac failure (n=3, 5%), cryptococcal meningitis (n=2, 3%), Kaposi’s sarcoma (n=2, 3%), Mycobacteria other than TB (n=2, 3%), epilepsy (n=1, 1.7%), Pneumocystis jirovecii (n=1, 1.7%), and sepsis (n=1, 1.7%). One hundred percent of deaths related to the AIDS diagnoses of cryptococcal meningitis, Kaposi’s sarcoma, Mycobacteria other than TB, and Pneumocystis jirovecii were in patients who had initiated ART within 14 days of screening. 

## Discussion

Our analysis reports on two major findings for immune compromised patients.

The first assesses guideline adherence for particularly immune compromised patients (CD4 < 50 cells/mm^3^) and demonstrates that expedited care is possible even in a busy South African setting serving a high percentage of immune compromised patients. Most (79%) received expedited ART well within the recommended 14 days at PHRU in Soweto, South Africa.

Second, we determined the effect of a maturing ART treatment program on mortality. Mortality was high in the first 3 to 6 months after starting ART. This high mortality is comparable to others reported in previous studies in Sub-Saharan Africa[19-22]. The proportion of deaths in the period 2004-2007 was significantly higher than that in 2008-2010. Variables predicting mortality in the early era were insignificant in the later era.

We observed that the mortality rate for immune compromised patients decreased as the ART program matured. It is plausible that treatment programs and healthcare professionals gain experience and skills in the management of advanced patients over time, potentially leading to better outcomes. This concurs with previously reported studies on predictors of mortality in HIV-infected patients in large ART treatment programs that show lower mortality outcomes as programs mature[4,23].

After controlling for age, baseline CD4 count, gender, time of initiating treatment and period, we found that previously hospitalized patients had a higher hazard of mortality. But when we determined predictors of mortality by period, hospitalization was predictive in the first period and none of the variables was predictive in the later period. As a treatment program matures, the risk of death declines. A possible explanation for this may be better management of patients as the treatment program matures leads to lower risk of death. Our findings concur with others reported elsewhere[4]. TB the main cause of death was similar between eras.

Because of limitations in our database, we could not include anemia, CDC staging, WHO staging and low BMI at baseline in our analysis, all of which are documented predictors of mortality. The lack of clinical disease data limits our ability to interpret our findings fully. It is possible that at the same CD4 value, patients in the earlier era were sicker (e.g. lower body mass index, more advanced clinical disease, lower haemoglobin) and it is unknown whether there would still be a difference in mortality between the eras after adjusting for clinical variables. Data was also not available for staff changes between the two eras. 

We found decreased mortality and a lower risk of death as an ART program matured in Soweto, South Africa. It is possible that this reflects an improvement in the management of patients presenting with advanced immune suppression over time 

Mortality decreased as the ART program matured in Soweto while time to initiation of treatment remained similar in both eras. Because ART guidelines were consistent during both eras, it is possible that with time, management of patients improved as expertise was gained. 
